# Comparative Transcriptome Analysis Reveals Hormone Signaling Genes Involved in the Launch of Culm-Shape Differentiation in *Dendrocalamus sinicus*

**DOI:** 10.3390/genes9010004

**Published:** 2017-12-22

**Authors:** Lingna Chen, Xiaojuan Guo, Yongzhong Cui, Xianggan Zheng, Hanqi Yang

**Affiliations:** Research Institute of Resources Insects, Chinese Academy of Forestry, Bailongsi, Panlong, Kunming 650233, China; chenln@caf.ac.cn (L.C.); lkygxj_417@163.com (X.G.); cafkmcyz@126.com (Y.C.); lkyzhxq_0926@aliyun.com (X.Z.)

**Keywords:** *Dendrocalamus sinicus*, comparative transcriptomic analysis, culm-shape differentiation, hormonal regulation

## Abstract

*Dendrocalamus sinicus* is a sympodial bamboo species endemic to Yunnan Province, China, and is the strongest bamboo species in the world. However, there is substantial variation in the basal culm shape, i.e., straight culm (SC) and bent culm (BC), among different *D. sinicus* as a result of genetic and growth factors. This study performed a comparative transcriptomic analysis of bamboo shoots of two variants at the early, mid-, and late shoot-development stages to examine the molecular basis of this variation. In total, 98,479 unigenes were annotated, of which 13,495 were differentially expressed in pairwise comparisons of the six libraries. More differentially expressed genes (DEGs) were involved in SC than in BC culm development. The DEGs between BC and SC were assigned to 108 metabolic pathways. The 1064 DEGs in early development might mainly control the launch of culm-shape differentiation. Sixty genes encoding components of hormone signaling pathways were differentially expressed between BC5 and SC5, indicating complex hormonal regulation of culm differentiation. The *AUX/IAA*, *ARF*, *PP2C*, *SnRK2*, and *ABF* genes involved in auxin and abscisic acid signaling played key roles. These results help us to understand the molecular mechanism of culm variation and other aspects of culm development in *D. sinicus*.

## 1. Introduction

Plant asymmetric growth or bending occurs in response to gravity, light, temperature, water, ions, and chemicals [1]. Plant stem bending is a complex biological process that has a significant impact on plant architecture and plant resource utilization. The response of plants to gravity is an important cue in plant stem bending [1]. An asymmetrical distribution of endogenesis hormone, starch accumulation in gravity perception, and the effects of the actin cytoskeleton on cell expansion all play important roles in the gravitropic response [2,3,4]. To elucidate the molecular mechanism of the response in higher plants, many mutants with aberrant stem or shoot gravitropism have been isolated from *Arabidopsis*, maize, sorghum, rice, and mulberry trees [2,3,5,6]. These results indicate that the physiological effects of auxins, gibberellins, and brassinosteroids are crucial for asymmetric plant growth [4,7,8]. They regulate stem development by modulating cell division and expansion, resulting in the gravitropic bending of roots and shoots, as well as other aspects of plant development [7,8]. However, the molecular mechanisms by which these factors regulate the development of stem traits remain poorly understood.

**Figure 1:**
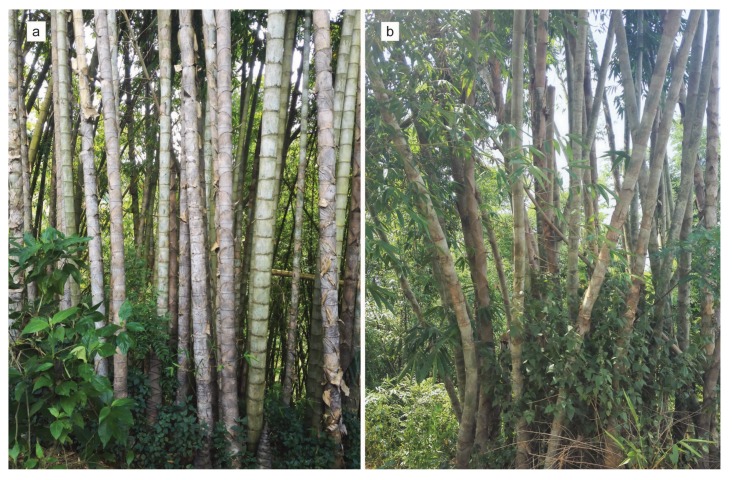
Vegetative forms of straight-culm (**a**) and bent-culm (**b**) *Dendrocalamus sinicus*.

*Dendrocalamus sinicus* L.C. Chia et J.L. Sun (hexaploid; 2n = 6x = 72) is a species of giant woody sympodial bamboo belonging to subfamily Bambusoideae in family Poaceae [9]. It is endemic to Yunnan, China. The species can reach 35 m in height, with culms 30 cm in diameter at breast height, and it is the strongest bamboo species documented [9,10]. The culm yield per unit area is 5–8 times as that of *Phyllostachys edulis*, which is a major economic bamboo species in China [11]. In its native habitat, *D. sinicus* is important as a raw material for furniture, construction, and industrial paper pulp. In nature, *D. sinicus* has two main stable culm-shape variants: the straight-culm (SC) and bent-culm (BC) types [12,13] (Figure 1). The ratio of fibrocyte length to diameter is 55.20–370.99 for straight-culm *D. sinicus*, reflecting elongate fibers, whereas the vascular strands and cells in the ground tissue of the bent-culm type show radial elongation, fibers, and vessel elements, with a relatively low ratio of fibrocyte length to diameter and short, wide fibers [14]. Molecular markers such as Random amplified polymorphic DNA (RAPD), Simple Sequence Repeat (SSR), *rpl*32-*trn*L, *rbc*L-*psa*I, and *trn*G-*trn*T indicate stable culm-shape variation in populations of *D. sinicus* [14,15,16]. Therefore, the straight- and bent-culm types of *D. sinicus* are an ideal comparison system for studying culm-shape development.

Regrettably, however, most molecular studies of woody bamboos have focused on flowering [17,18,19], rhizomes [20], bud burst and rattling growth [21,22,23,24,25], and primary thickening growth [26]. Little research has examined the molecular mechanism of culm-shape variation in *D. sinicus* and other bamboos. Comparative transcriptome analysis is a promising, powerful approach for discovering the molecular basis of specific biological events due to its high-throughput, accuracy, and reproducibility [27,28]. Here, we report a detailed comparative transcriptomic analysis of culm types SC and BC of *D. sinicus* at the early, mid-, and late shoot-development stages. This study explored the mechanism of culm-shape differentiation and identified significant differences in the transcriptomes of SC and BC *D. sinicus*. As a natural reference system, this difference in development between the two culm types provides an opportunity to study the molecular mechanism that controls culm-shape differentiation in *D. sinicus*. This study also enables a comprehensive understanding of culm development in woody bamboo.

## 2. Materials and Methods 

### 2.1. Plant Materials 

Two culm types, SC and BC *D. sinicus* from Ximeng (99°32′35 E, 22°43′51 N) and Menglian (99°39′53 E, 22°26′61 N) Counties, Yunnan Province, respectively, were sampled and used for the transcriptome analysis. The culm shape of *D. sinicus* stabilizes during the first month of bamboo shoot growth, when the plants are about 5 m tall and culm-shape differentiation can be seen visually [12]. The variation in culm shape is mainly observed at basal internodes within 1–3 m of the ground. Bent-culm type *D. sinicus* is characterized by nodal distortion within the basal portion (1–3 m) of the culm, which presents as shortening, swelling, and skewing, making the processing and utilization of bamboo difficult. On the other hand, straight-culm type *D. sinicus* grows normally. The culm taper is small, the height under the branches exceeds 12 m, and the nodal wall thickness at about 4 m off the ground can reach 2 cm. In June 2016, nine bamboo shoots with similar ground diameters were selected from each type and observed morphologically from Day 0, when the shoots were unearthed. Each type was sampled with three biological replicates at the early (5-day-old bamboo shoots), middle (15-day-old), and late (30-day-old) stages of the culm shape changes. The node and internode at the 15th node from the unearthed shoots were cut, transferred to liquid nitrogen immediately, and stored at –80 °C until RNA extraction. The samples of stages the 5-, 15-, and 30-day-old BC and SC shoots were named BC5, BC15, BC30, SC5, SC15, and SC30, respectively.

### 2.2. RNA Isolation, Library Construction, and Sequencing

Total RNA from each sample was isolated separately using a TransZol Up Plus RNA Kit (TransGen Biotech, Beijing, China) according to the manufacturer’s protocol. RNA integrity was evaluated in a 1.0% agarose gel with GelStain (TransGen Biotech, Beijing, China). Then, the quality and quantity of total RNA was assessed using a NanoDrop 2000 spectrophotometer (Thermo Fisher Scientific, Waltham, MA, USA) and Qubit (Thermo Fisher Scientific, Waltham, MA, USA). RNA samples from the three individuals at each stage and for each type were pooled in equal amounts to generate one mixed sample. The six mixed RNA samples were subsequently used for cDNA library construction and paired-end sequencing, which was performed on the Illumina HiSeq2000 system.

### 2.3. De Novo Transcriptome Assembly and Functional Annotation

The raw reads from transcriptome sequencing were filtered by removing adaptor sequences, reads containing poly-N, and low-quality reads (Q < 20). The clean reads were assembled into contigs using the program Trinity [29]. The transcriptome data in this study were deposited in the National Center for Biotechnology Information (NCBI) Short Read Archive (SRA) database under the accession PRJNA418355. This pipeline performs annotation to the NCBI non-redundant (Nr) database and Kyoto Encyclopaedia of Genes and Genomes (KEGG) pathway annotation.

### 2.4. Functional Analysis of Differentially Expressed Genes 

The transcript abundance was quantified using RSEM software [30]. Then, the differentially expressed genes (DEGs) between pairs of samples were detected using the DESeq package [31], with a *p*-value ≤0.01, a False Discovery Rate (FDR) <0.01, and a fold change >2. The KEGG Pathway enrichment of DEGs was evaluated using a hypergeometric test with a significance threshold of 0.05 after the Benjamini and Hochberg FDR correction in KOBAS (2.0) [32].

### 2.5. Validation of DEGs Using qRT-PCR 

To validate the results of transcriptome sequencing, the expression of 10 DEGs involved in plant hormone signal transduction and phenylpropanoid biosynthesis was investigated using the CFX96 Real-Time PCR Detection System (Bio-Rad, Hercules, CA, USA). Primers for quantitative real-time PCR (qRT-PCR) were designed using Primer 3 [33] (Table 1). cDNA was synthesized from 10 μg of total RNA using TransScript II One-Step gDNA Removal and cDNA Synthesis SuperMix (TransGen Biotech, Beijing, China) as per the manufacturer’s protocol. *GAPDH* was used as an internal control gene for normalization. The amplifications were performed using the SYBR Green I method with TransStart Top Green qPCR SuperMix (TransGen Biotech, Beijing, China) according to the manufacturer’s instructions. The relative expression levels of the selected unigenes were analyzed using the 2^–ΔΔC^_T_ method [34]. This experiment was carried out in three biological replicates, each with three technical replicates.

## 3. Results

### 3.1. Transcriptome Sequencing and De Novo Assembly of D. sinicus

To understand the transcriptional changes that may be involved in regulating culm-shape differentiation, BC and SC were sampled from 5-, 15-, and 30-day-old bamboo shoots (representing the early, mid-, and late shoot-development stages, respectively) for high-throughput sequencing. In total, 352 million raw reads were generated from the six libraries (BC5, BC15, BC30, SC5, SC15, and SC30), and 340 million (97%) high-quality (*Q* > 20) reads were obtained after removing lower-quality and adapter-contaminated reads (Table 2). The average 53.1% GC content of *D. sinicus* unigenes was in the range of GC levels of coding sequences in monocots [35].

**Figure 2:**
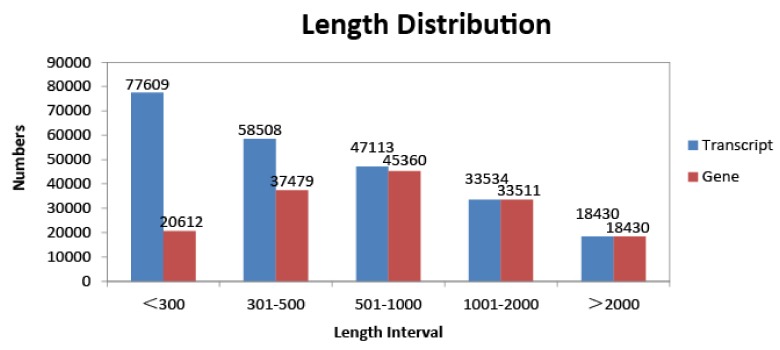
Length distribution of *D. sinicus* transcripts and unigenes.

The de novo transcriptome assembly generated 155,392 unigenes, with a maximum length of 15,773 bp and a mean length of 989 bp, of which 103,451 unigenes (66.6%) had lengths of 200–1000 bp, 33,511 unigenes (21.6%) had lengths of 1000–2000 bp, and 18,430 unigenes (11.9%) had lengths >2000 bp (Figure 2). The transcriptome was annotated using a search against the NCBI Nr, Gene Ontology (GO), and KEGG databases; 98,479 unigenes had at least one hit in these databases.

### 3.2. Differentially Expressed Genes Analysis during Culm Development

**Figure 3:**
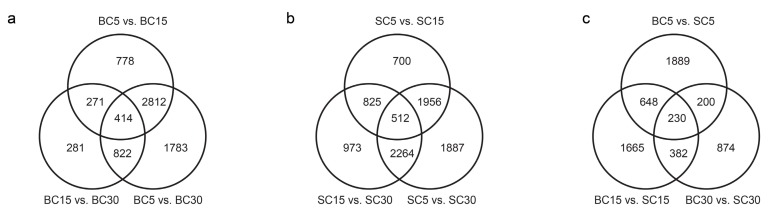
Overview of the serial analysis of differentially expressed genes (DEGs) identified by pairwise comparisons of the six transcriptomes: SC5, SC15, SC30, BC5, BC15, and BC30. (**a**) Venn diagram of the DEGs in bent culm (BC) at the three stages; (**b**) Venn diagram of the DEGs in straight culm (SC) at the three stages; (**c**) Venn diagram of the DEGs between BC and SC at the three stages.

The expression levels of the genes in the two culm types of *D. sinicus* were examined at three growth stages, and DEGs were identified by pairwise comparisons of the six libraries (Figure 3). Comparisons of the three BC stages identified 4275, 1788, and 5831 DEGs in the pairs BC5 vs. BC15, BC15 vs. BC30, and BC5 vs. BC30, respectively (Figure 3a). For SC, 3993, 4574, and 6619 DEGs were identified in the pairs SC5 vs. SC15, SC15 vs. SC30, and SC5 vs. SC30, respectively (Figure 3b). The total number of DEGs across the three stages was higher in SC than in BC, with 9117 and 7161 DEGs, respectively. Comparisons of BC and SC at the same stages identified 2967, 2925, and 1686 DEGs in the 5-, 15-, and 30-day-old bamboo shoots, respectively (Figure 3c). Fewer DEGs were detected in the same-stage comparisons between the two culm types of *D. sinicus* than in the same-type comparisons at different stages. In total, 13,495 genes were differentially expressed in BC and SC at the three stages. Of these DEGs, 5888 were significantly regulated in both types during culm development; 1273 and 3229 genes were differentially regulated in BC and SC, respectively, indicating that more DEGs are involved in culm development in SC.

The KEGG enrichment analysis of DEGs assigned them to 108 KEGG pathways, including plant hormone signal transduction, phenylpropanoid biosynthesis, the citrate cycle, and oxidative phosphorylation (see Supplementary File S1, in which the details of significantly enriched genes in every KEGG pathway are shown). Table 3 shows the five largest pathways at every stage. At the three development stages, there were 1064, 1090, and 500 DEGs, respectively, between BC and SC. The plant hormone signal transduction pathways included the most DEGs between BC and SC in the 5-day-old bamboo shoots. The expression of 44 of 60 DEGs was higher in BC5 than in SC5. The number of DEGs involved in the phenylpropanoid biosynthesis pathway was always high, with 39, 50, and 40 DEGs, respectively, assigned to the pathway in 5-, 15-, and 30-day-old bamboo shoots. Of these, the expression level of 33, 15, and 22 DEGs was higher in BC, indicating a dramatic difference in lignin biosynthesis throughout the course of culm development.

### 3.3. DEGs Involved in Plant Hormone Signal Transduction

Plant hormones are involved in many different processes throughout the life of a plant, including growth, development, and senescence [36]. In this study, 428 DEGs assigned to hormone signal transduction, hormone biosynthesis pathways, hormone responsive proteins, and hormone transporter proteins were identified; 179, 108, 65, 37, 12, and 7 of them were involved in auxin, ethylene, abscisic acid (ABA), gibberellin (GA), jasmonic acid (JA), and brassinosteroid (BR) pathways, respectively. Of these, there were respectively 60, 31, and 14 DEGs between BC and SC at 5, 15, and 30 days of development. Most of them showed higher expression in BC compared with SC at the same development stage. As with the ABA signaling pathway, 15 protein phosphatase 2C (*PP2C*) and 6 ABRE Binding Factor (*ABF*) transcription factors had higher expression in BC. The same was true for the gibberellin receptor *GID1* and *DELLA* gene in the GA signaling pathway and for jasmonic acid–amido synthetase (*JAR1*) and transcription factor *MYC2* genes in the JA signaling pathway. Nevertheless, there were some exceptions; most of the auxin-responsive protein *AUX/IAA* and all *SAUR* families involved in auxin signal transduction were generally higher in SC.

**Figure 4:**
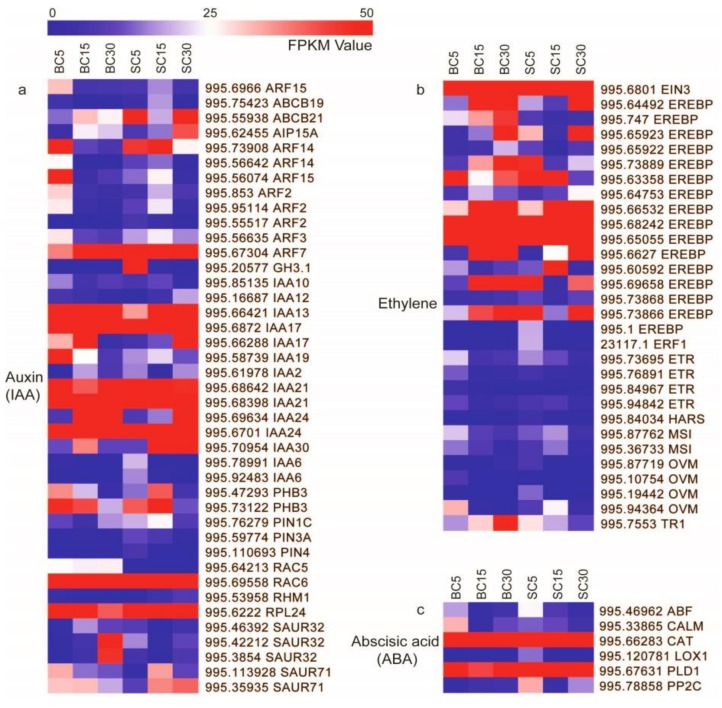
Heatmap of differentially expressed genes assigned to hormone signal transduction pathways in the six *D. sinicus* transcriptomes: BC5, BC15, BC30, SC5, SC15, and SC30. DEGs related to (**a**) auxin, (**b**) ethylene, and (**c**) abscisic acid. FPKM (fragments per kilobase of exon per million mapped reads) values range from 0 to 50.

Auxin, the first plant growth hormone discovered, plays a pivotal role in plant growth, including the development of roots, shoots, flowers, and fruit [37]. There were 179 DEGs that encoded genes such as auxin response factor (*ARF*), *AUX/IAA*, *SAUR*, *PIN*, *ABC transporter*, etc. Of these, 77 DEGs with similar expression patterns in BC and SC with culm development might participate in culm elongation. The genes in the upregulated groups with the highest expression were *AUX/IAA*, including the *IAA1-like* (Cluster-995.67344), *IAA3-like* (Cluster-995.68181), *IAA17-like* (Cluster-995.66645), and *IAA30-like* (Cluster-995.67202) genes. The gene expression increased gradually with development of the bamboo shoot. Another 56 DEGs with different expression patterns (Figure 4) should not be ignored in culm-shape differentiation of *D. sinicus*. The DEGs included *AUX/IAA*, *ARF*, *SAUR*, and *CH3*. Of these genes, 24 and 11 were *AUX/IAA* and *ARF* family members, respectively. Of these, the *auxin-repressed 12.5 kDa protein-like* (Cluster-995.68211) gene had the highest expression, followed by the *ARP13-like* (Cluster-995.66421) and *IAA17-like* genes (Cluster-995.68720).

The expression of genes in the ABA signaling pathway was also compelling. There were 15 *PP2C*, 15 sucrosenon-fermenting1-related protein kinase *SnRK2*, 6 *ABF*, and 5 ABA receptor *PYR* genes with differential expression. Three abscisic stress ripening (*ASR*) genes, Cluster-995.67334, Cluster-995.67322, and Cluster-995.67324, had the highest expression. They were sharply up-regulated on the 15th day of shoot development and maintained high expression in later development stages. In terms of the GA, ethylene, and JA signaling pathways, the regulated expression of *GID1*, *DELLA*, *ETR*, *JAR1*, *MYC2*, etc. indicated that they are also involved in shoot development.

### 3.4. DEGs Involved in Phenylpropanoid Biosynthesis

**Figure 5:**
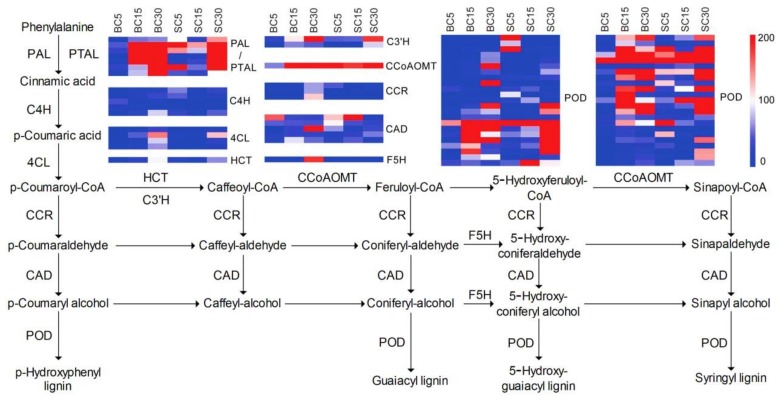
Expression patterns of the expressed genes assigned to lignin biosynthesis in the six *D. sinicus* transcriptomes: BC5, BC15, BC30, SC5, SC15, and SC30. Completed according to KEGG source record: ko00940. All metabolic enzymes predicted in the *D. sinicus* transcriptome are marked in the pathway. FPKM values range from 0 to 200. PAL: phenylalanine ammonia-lyase; PTAL: phenylalanine/tyrosine ammonia-lyase; C4H: cinnamate 4-hydroxylase; 4CL: 4-coumarate–CoA ligase; HCT: shikimate *O*-hydroxycinnamoyltransferase; C3′H: coumaroylquinate(coumaroylshikimate)3′-monooxygenase; CCoAOMT: caffeoyl-CoA *O*-methyltransferase; CCR: cinnamoyl–CoA reductase, CAD: cinnamyl–alcohol dehydrogenase; F5H: ferulate-5-hydroxylase; POD: peroxidase.

Phenylpropanoid-based polymers that have extensive physiological activity contribute substantially to regulating plant growth and defence against disease [38,39]. In our study, from the annotated 98,479 unigenes, 394 unigenes encoding 16 key enzymes were identified as involved in phenylpropanoid biosynthesis. Among these, 93 unigenes encoding 15 key enzymes showed different expression levels at different stages in BC and SC. They were mainly involved in lignin synthesis, and included *p*-hydroxyphenyl lignin (H type), guaiacyl lignin (G type), 5-hydroxyguaiacyl lignin, and syringyl lignin (S type) (Figure 5), suggesting obvious differences in lignin synthesis between BC and SC plants. Of the 93 DEGs encoding peroxidase, 47 had high expression, which varied greatly throughout development. The fragments per kilobase of transcript per million mapped reads (FPKM) of Cluster-995.67494 exceeded 4000 in SC15. The expression of the unigenes decreased throughout BC development, whereas it increased initially and then decreased with development in SC. As the first step, *PATL* had continuous high expression with culm development. The FPKM value of Cluster-995.66569 exceeded 4000 in BC30 and SC30.

### 3.5. Verification of the Gene Expression through qRT-PCR

**Figure 6:**
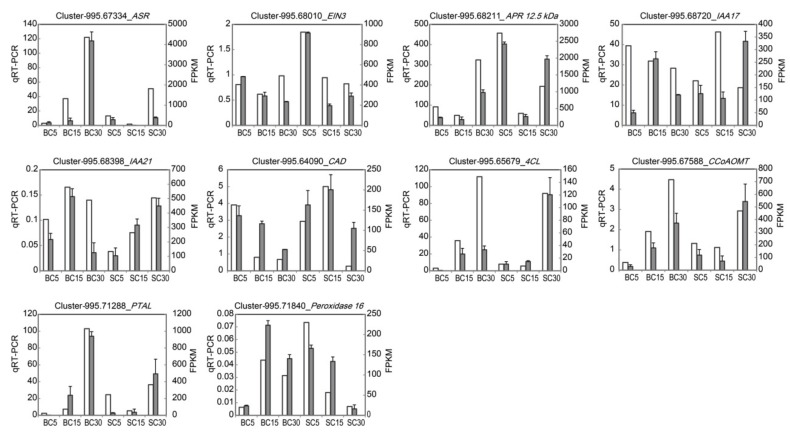
Expression of 10 selected genes as determined by qRT-PCR in comparison with the transcriptome results. The qRT-PCR values for each gene are the means ± SD of three biological replicates, with three technical replicates per experiment. The gene names and primers used for qRT-PCR analysis are shown in Table 1. The white bars indicate FPKM values as determined by transcriptome sequencing, and the grey bars show the expression as determined by qRT-PCR. *ASR*: abscisic acid stress ripening protein; *EIN3*: ethylene-insensitive protein 3; *APR 12*.*5 kDa*: auxin-repressed 12.5 *kDa* protein-like; *IAA17*: auxin-responsive protein IAA17-like; *IAA21*: auxin-responsive protein IAA21; *CAD*: cinnamyl–alcohol dehydrogenase; *4CL*: 4-coumarate–CoA ligase; *CCoAOMT*: caffeoyl–CoA *O*-methyltransferase; *PTAL*: phenylalanine/tyrosine ammonia-lyase.

The results of the DEG analysis obtained from the transcriptome data were validated in a biologically independent manner using qRT-PCR. Ten plant hormone signal transduction and phenylpropanoid biosynthesis DEGs were selected. Of these, nine showed similar expression patterns in the qRT-PCR data and the transcriptome results (Figure 6), suggesting the consistency of the results.

## 4. Discussion

### 4.1. Transcriptome Changes in Culm-Shape Differentiation of D. sinicus

The culm development of *D. sinicus* is regulated by complex, endogenous factors according to a transcriptome analysis of different internodes originating from the same bamboo plant [22]. However, the study elucidated the molecular processes involved in culm elongation of straight-culm *D. sinicus* rather than differentiation of two culm-shape variants. To understand molecular mechanisms controlling the culm-shape differentiation, we sequenced and annotated the transcriptome of three critical culm-development stages from the bent-culm *D. sinicus* and the straight-culm *D. sinicus*. We generated 177 and 163 million paired end reads from types BC and SC, respectively. Unfortunately, de novo transcriptome assembly of *D. sinicus* had the lower mapped reads about 70% because of suboptimal parameter settings for polyploids in Trinity program [40]. More than 88 percent of unmapped reads matched to monocots such as *Oryza sativa*, *Setaria italic*, and *Zea mays*. The 98,479 annotated genes constituted 1.5 times the number of annotated genes in the transcriptome analysis of culm elongation of *D. sinicus* [22]. A total of 13,495 DEGs showed an enormous change during shoot development in the two types of *D. sinicus*. This is the first report of the molecular mechanism of culm-shape variation in woody bamboo obtained by contrasting a natural reference system. The greater number of DEGs involved in culm development in SC provided valuable clues for further elucidation of the mechanisms regulating culm development [36]. Although the draft genome of *Phyllostachys heterocycla* [41] and transcriptome data of other bamboo species have been reported [18,22,42], our study was the first to examine culm-shape differentiation of *D. sinicus*.

Based on the comparative transcriptome analysis, the two types examined in our study showed distinct differences related to culm development. There were significantly more DEGs between BC and SC in the 5- and 15-day-old bamboo shoots, indicating that culm shape differentiates at an early development stage in *D. sinicus*. The Pearson correlation coefficients in the analysis of gene expression levels between BC and SC were 0.575, 0.618, and 0.69 at days 5, 15, and 30, respectively, which strengthened this result. The KEGG pathways analysis found that most of the DEGs were involved in plant hormone signal transduction and phenylpropanoid biosynthesis, suggesting that they have prominent roles in culm development in *D. sinicus*.

### 4.2. DEGs Involved in Plant Hormone Signal Transduction during Culm-Shape Differentiation

Plant stem or culm development is a complex biological process, and many genes participate in the hormone signal transduction and secondary metabolism that are involved in plant stem bending and related traits [43,44]. The present comparative transcriptome analysis of the two types of *D. sinicus* supports this view. In this study, the differential expression of 60 related genes in early development indicated that they played important roles in launching culm-shape differentiation in *D. sinicus*.

Auxin, which was the first plant endogenous hormone discovered, promotes cell division to adjust stem growth and development by regulating the cell cycle, enabling extension of the cell wall, and inducing the synthesis of RNA and proteins [37]. The asymmetrical distribution of IAA in tissues is the main cause of plant organ bending [37]. In *Arabidopsis thaliana*, knockout or suppression of *AUX1* and *PIN2*, two IAA carriers, completely blocked or altered root gravitropism bending [8]. Overexpression of rice *OsIAA1*, *OsIAA3*, and *OsIAA4* also revealed that they are involved in root, stem, and leaf architecture [45]. Very recent reports suggest that auxin signaling genes play a central role in shoot growth in woody bamboo [26,43,46]. In our study, gene families participating in auxin synthesis and signal transduction were obtained from the transcriptomes of two types of *D. sinicus*. Among 56 DEGs that had markedly different expression patterns in BC and SC, 24 and 11 were *AUX/IAA* and *ARF* family members, respectively, including *IAA1* (Cluster-995.83130), *IAA4* (Cluster-995.72629), *IAA7* (Cluster-995.113198), *IAA14* (Cluster-995.73908), *IAA17* (Cluster-995.66288 and Cluster-995.68720), and *IAA19* (Cluster-995.58739). Their role in root, hypocotyl, shoot, and inflorescence gravitropism bending has been well documented in *Arabidopsis* mutants and rice [45,47,48]. The role of homologous genes in culm-shape differentiation of *D. sinicus* should be noted. In addition, *ARF7* (Cluster-995.67304), *IAA2* (Cluster-995.61978), *SAUR32* (Cluster-995.46392), and *RAC6* (Cluster-995.69558), which had completely opposite expression patterns, might have important roles in culm differentiation.

However, culm development is not the result of the action of a single hormone, but involves complex cross-talk among plant hormones [44]. In this study, numerous genes in the ABA signaling pathway showed obvious differences in expression between the two types of *D. sinicus*. They included 15 *PP2C* and 15 *SnRK2* gene family members, which are important ABA signal mediators [49]. Additionally, higher expression of two important GA signaling genes, *GID1* and *DELLA*, in BC5 compared with SC5 suggested that culm-shape differentiation in *D. sinicus* depends on GA signal transduction. Several studies have suggested that the interaction of auxin with GA and BRs is involved in the gravitropic response of shoots and roots [50,51]. These findings suggest that culm-shape differentiation in *D. sinicus* is dependent on a hormone-mediated signal transduction process, especially involving auxin and ABA.

### 4.3. DEGs Involved in Lignin Biosynthesis during Culm-Shape Differentiation

Our transcriptomic data revealed differences in the expression of lignin synthesis-related genes during shoot development. Therefore, lignin synthesis probably facilitates the construction of structural components during bamboo culm development, including culm elongation [46,52,53]. In this study, 16 annotated enzymes involved in the biosynthesis of monolignols, including H-, G-, and S-type monolignols, played roles in the formation and development of the bamboo [38]. The differences in the expression of genes encoding key enzymes in the pathways hinted at variation in lignin biosynthesis in *D. sinicus* (Figure 5). *PAL*/*PTAL* catalyzes the first step in the phenylpropanoid biosynthesis pathway, and *PAL*/*PTAL* expression and abundance directly affect the synthesis of lignin [39]. In our study, six DEGs between BC and SC encoding PAL/PTAL were up-regulated as culm development proceeded in BC, whereas only Cluster-995.68104 and Cluster-995.68617 were up-regulated in SC, although the greatest expression was seen in BC30 and SC30. In the 5-day-old bamboo shoots, the expression of 6 *PAL*/*PTAL* was much higher in SC compared with BC, which was opposite the expression in the 15-day-old bamboo shoots, revealing greater changes in lignin biosynthesis in BC occur during the early stage of culm development. The contribution of the corresponding enzymes leading to different monolignol compositions and resulting in various ratios of different units of lignin have been summarized, and the effects of the corresponding genes have been analyzed in detail [38,54,55]. We found that *CCoAOMT* and *PAL* were expressed similarly. Higher expression of these genes during the early development stage suggested that there is more G-type lignin in BC. *CCoAOMT* suppression in *Medicago sativa*, *Zea mays*, and *Pinus radiate* causes a reduction in G-type lignin [56,57,58,59], and the RNAi-mediated suppression of *PAL* in *Salvia miltiorrhiza* also led to abnormal phenotypes, including stunted growth, delayed root formation, and altered leaves [60]. Further research is needed to understand the function of *PAL* and *CCoAOMT* in *D. sinicus*. And cellulose biosynthesis genes, such as *cellulose synthase* (*CesA*) and *cellulose synthase-like* (*CSL*) genes, were also found to be expressed contribution to rapid culm development of *D. sinicus*, as previously reported in *Dendrocalamus hamiltonii* [25].

Overall, culm-shape differentiation of *D. sinicus* is a complex developmental process that depends on the balanced expression of genes within a complex network. The launch of culm-shape differentiation in *D. sinicus* might be controlled mainly by the genes in hormone transduction pathways, whereas culm development might be mainly controlled by the genes in phenylpropanoid biosynthesis pathways [22,43]. Further studies of the functions of these potential candidate genes might help us to understand culm-shape differentiation in *D. sinicus*.

## Figures and Tables

**Table 1:** Primers for quantitative PCR validation of the RNA-seq data.

Gene ID	Description	Primer	
		Forward (5′-3′)	Reverse (5′-3′)
Cluster-995.67334	*ASR*	GCGAGAACGAGTACGAGAGGT	GATCTCCTCCGCGATCTTGTG
Cluster-995.68010	*EIN3*	CGGTCGAAGCGGACCTTCTC	GCAGGACGGCATCCTCAAGTA
Cluster-995.68211	*ARP 12.5 kDa*	GTACTCGGACAGTGCAGGCTAGA	TGGTGGCGGCCTTGCGGAGCTT
Cluster-995.68720	*IAA17*	CTCCGGTTCATGTCCTGCTGCT	AACGCGGCGGTCCGGCGACTCG
Cluster-995.68398	*IAA21*	CACAGGAGCGGGACTACATC	CGCCCTTGGGCAGCAG
Cluster-995.64090	*CAD*	GATTCGTCCGGTGTCCTCTC	GGGAACAACGGGGTACAGAG
Cluster-995.65679	*4CL*	GGGGACTTCACCAGCAAGTT	GCACACTGGAGACATCGGTT
Cluster-995.67588	*CCoAOMT*	TAGTTGTCCTTGTCGGCGTC	TCAACCGCGAGAACTACGAG
Cluster-995.71288	*PTAL*	AGAGCTCCGTCAAGAACTGC	TCTGCATCAGCGGGTAGTTG
Cluster-995.71840	*Peroxidase 16*	TGGTAGTAGCCGTTGTCGAAC	GCGGCTGTACACGTTCAAG

**Table 2:** Overall assembly statistics for the *D. sinicus* transcriptomes.

	BC5	BC15	BC30	SC5	SC15	SC30
Number of raw reads	58,731,962	65,460,126	59,431,136	54,367,904	56,408,792	57,867,298
Number of clean reads (%)	56,597,392 (96.37%)	63,275,200 (96.66%)	57,515,272 (96.78%)	53,084,380 (97.64%)	55,287,738 (98.01%)	54,692,784 (94.51%)
Q20 (%)	97.29	97.49	97.4	97.29	97.40	97.63
Mapped reads (%)	39,026,306 (68.95%)	44,519,052 (70.36%)	39,786,978 (69.18%)	36,775,186 (69.28%)	39,279,750 (71.05%)	37,541,394 (68.64%)
GC (%)	52.01	52.31	54.27	53.44	54.07	51.24

Q20 (sequencing base calls with an error rate of less than 1%); BC5 (5 day old bent culm); BC15 (15 day old bent culm); BC30 (30 day old bent culm); SC5 (5 day old straight culm); SC15 (15 day old straight culm); SC30 (30 day old straight culm).

**Table 3:** Kyoto Encyclopaedia of Genes and Genomes (KEGG) classification of DEGs between the two types of *D. sinicus* at the same culm-development stages.

Development Stages	KEGG Categories	Rich Factor	Corrected *p*-Value	No. of Unigenes
BC5 vs. SC5	Plant hormone signal transduction	0.089	0.002	60
	alpha-Linolenic acid metabolism	0.158	0.002	18
	Stilbenoid, diarylheptanoid, and gingerol biosynthesis	0.222	0.002	12
	Phenylalanine metabolism	0.150	0.002	19
	Phenylpropanoid biosynthesis	0.099	0.003	39
BC15 vs. SC15	Phenylpropanoid biosynthesis	0.127	5.52 × 10^−6^	50
	Cyanoamino acid metabolism	0.169	1.94 × 10^−4^	23
	Steroid biosynthesis	0.197	0.004	13
	Citrate cycle (TCA cycle)	0.119	0.021	21
	Glycolysis/Gluconeogenesis	0.087	0.038	40
BC30 vs. SC30	Phenylpropanoid biosynthesis	0.102	1.23 × 10^−11^	40
	Flavonoid biosynthesis	0.137	0.001	10
	Flavone and flavonol biosynthesis	0.455	0.001	5
	Glyoxylate and dicarboxylate metabolism	0.082	0.001	16
	Oxidative phosphorylation	0.058	0.001	25

## Supplementary Materials:

The following are available online at www.mdpi.com/2073-4425/9/1/4/s1. Supplementary File S1: Significantly enriched genes in every KEGG pathways.
